# Cardiac Magnetic Resonance Imaging for Nonischemic Cardiac Disease in Out-of-Hospital Cardiac Arrest Survivors Treated with Targeted Temperature Management: A Multicenter Retrospective Analysis

**DOI:** 10.3390/jcm10040794

**Published:** 2021-02-16

**Authors:** Sang-Min Kim, Chun-Song Youn, Gun-Tak Lee, Tae-Gun Shin, June-Sung Kim, Youn-Jung Kim, Won-Young Kim

**Affiliations:** 1 Department of Emergency Medicine, Ulsan University College of Medicine, Asan Medical Center, Seoul 05505, Korea; swdarkhorse@gmail.com (S.-M.K.); jsmeet09@gmail.com (J.-S.K.); yjkim.em@gmail.com (Y.-J.K.); 2 Department of Emergency Medicine, Seoul St. Mary’s Hospital, College of Medicine, The Catholic University of Korea, Seoul 07345, Korea; ycs1005@catholic.ac.kr; 3 Department of Emergency Medicine, Sungkyunkwan University School of Medicine, Samsung Medical Center, Seoul 06351, Korea; guntak.lee@samsung.com (G.-T.L.); taegunshin@skku.edu (T.-G.S.)

**Keywords:** cardiac MRI, cardiac arrest, targeted temperature management

## Abstract

(1) Background: Cardiac magnetic resonance (CMR) imaging is an emerging tool for investigating nonischemic cardiomyopathies and cardiac systemic disease. However, data on the cardiac arrest population are limited. This study aimed to evaluate the usefulness of CMR imaging in out-of-hospital cardiac arrest (OHCA) survivors treated with targeted temperature management (TTM). (2) Methods: We conducted the retrospective observational study using a multicenter registry of adult non-traumatic comatose OHCA survivors who underwent TTM between January 2010 and December 2019. Of the 949 patients, 389 with OHCA of non-cardiac cause, 145 with significant lesions in the coronary artery, 151 who died during TTM, 81 without further evaluation due to anticipated poor neurological outcome, and 51 whose etiology is underlying disease were excluded. In 36 of the 132 remaining patients, the etiologies included variant angina, long QT syndrome, and complete atrioventricular block in ancillary studies. Fifty-six patients were diagnosed idiopathic ventricular fibrillation without CMR. (3) Results: CMR imaging was performed in the remaining 40 patients with cardiac arrest of unknown cause. The median time from cardiac arrest to CMR imaging was 10.1 days. The CMR finding was normal in 23 patients, non-diagnostic in 12, and abnormal in 5, which suggested non-ischemic cardiomyopathy but did not support the final diagnosis. (4) Conclusions: CMR imaging may not be useful for identifying unknown causes of cardiac arrest in OHCA survivors treated with targeted temperature management without definitive diagnosis even after coronary angiography, echocardiography, and electrophysiology studies. However, further large-scale studies will be needed to confirm these findings.

## 1. Introduction

Out-of-hospital cardiac arrest (OHCA) is one of the leading causes of death in industrialized countries [1]. Targeted temperature management (TTM) is generally recommended for patients who are comatose after post-cardiac arrest [2]. Identifying and treating the cause have also been emphasized to be major issues in the recent guidelines of the International Liaison Committee on Resuscitation [2]. Although ischemic heart disease is well known to account for most cases with cardiac origin of arrest, the proportion of cases of other causes, which can be divided into structural (non-ischemic cardiomyopathy, sarcoidosis, amyloidosis, and myocarditis) or electrical abnormalities (long QT syndrome, Brugada syndrome, and polymorphic ventricular tachycardia), is not negligible [3]. However, the cause of OHCA can remain uncertain even after echocardiography and coronary angiography. A previous study reported that approximately 40% of cases remain without an overt diagnosis despite vigorous efforts to elucidate the causes of cardiac arrest [4]. It is still difficult to elucidate the etiology of cardiac arrest without significant coronary artery lesion for physicians. 

Cardiac magnetic resonance (CMR) imaging is helpful for evaluating various cardiac disorders, as it provides information about heart function, structure, tissue characterization with better accuracy, and reproducibility, as compared with echocardiography [5,6]. CMR imaging is recommended as an alternative cardiac imaging modality for patients with non-diagnostic echocardiographic studies in the European Society of Cardiology guideline [7]. CMR imaging also provides crucial information for risk stratification of sudden cardiac death in non-ischemic cardiomyopathy with late gadolinium enhancement sequence [8,9]. Additionally, CMR can be of great value in evaluation of arrhythmogenesis in sudden cardiac death [10]. Knowledge from investigations using CMR imaging for the etiology of sudden cardiac death is limited and inconsistent. Some studies found an additional diagnostic value of CMR imaging in patients with cardiac arrest, whereas others showed only subtle CMR imaging findings [11,12,13,14]. Moreover, most studies focused on sudden cardiac death included patients with alert mentality after the return of spontaneous circulation. However, patients treated with TTM have different characteristics, and there is a paucity of data from these population.

The aim of this study was to evaluate the usefulness of CMR imaging in OHCA survivors with inconclusive coronary angiography findings who were treated with TTM.

## 2. Material and Methods

### 2.1. Study Design and Patients

This retrospective observational registry-based study was conducted at a 3 tertiary care university-affiliated teaching hospital in Korea. The data of 949 consecutive adult (≥18 years) patients with non-traumatic OHCA treated with TTM between January 2010 and December 2019 were extracted from prospectively collected OHCA registry data [15,16,17,18]. The number of patients from each hospital is as follows: 328, 360, and 261, respectively. OHCA cases with presumed cardiac or unknown cause were included. Non-cardiac causes of arrest, such as drug intoxication, hanging, asphyxia, and drowning, were excluded. No further evaluation was performed for patients with an anticipated poor prognosis or short life expectancy and a known underlying cardiac disease suspected as the cause of arrest. Patients with a culprit lesion proven on coronary angiography were also excluded. CMR imaging was performed in the patients whose etiology of arrest was not proven by other studies, including ergonovine stress test or electrophysiology study. Echocardiography was performed before CMR imaging in all the enrolled patients.

The Institutional Review Board of the University of Ulsan College of Medicine reviewed and approved the study protocol (IRB No. 1052), and informed consent was waived because of the retrospective nature of the study.

### 2.2. Management and Data Collection

During the study period, all OHCA patients received cardiopulmonary resuscitation and post-resuscitation care, including TTM and coronary reperfusion, according to the then-current advanced cardiac life support guidelines [19,20]. TTM was performed for all unconscious patients using cooling devices such as the Blanketrol II (Cincinnati Subzero Products, Cincinnati, OH, USA), Arctic Sun Energy Transfer Pad (Medivance Corp., Louisville, CO, USA), or an endovascular cooling device (Thermoguard; ZOLL Medical Corporation, Chelmsford, MA, USA). The target temperature (33 °C or 36 °C) was maintained for 24 h. After 24 h, patients were rewarmed at a rate of 0.25 °C/h following maintained normothermia until 72 h from ROSC. The temperature was monitored using an esophageal or rectal temperature probe. We used propofol, benzodiazepine, and opioids for sedation and analgesia. If necessary, a neuromuscular blocking agent was administered to control shivering. All patients received standard intensive care according to institutional protocols.

Demographic and clinical data such as age, sex, witnessed cardiopulmonary resuscitation (CPR), bystander CPR, time from cardiac arrest to CMR imaging, medical history (previous cardiac arrest, previous myocardial infarction, angina, arrhythmia, heart failure, and hypertension), electrocardiographic findings, initial rhythm at the scene, shock at the scene, and initial rhythm at the emergency department were collected from the electronic medical records of registry-enrolled patients. There were no missing values to manage.

### 2.3. Echocardiographic Analysis

Transthoracic echocardiography (TTE) was performed before CMR imaging in all the enrolled patients by experienced cardiologists and/or sonographers after TTM. Complete standard TTE was performed in accordance with the clinical laboratory practice and international recommendations [21]. Left ventricular (LV) end-diastolic (EDV) and end-systolic volumes (ESV) and biplane ejection fractions were measured from the 4- and 2-chamber views using the Simpson or Teichholz method. Two investigators blinded to the patient’s clinical history and data analyzed all examination results. All anatomical and functional parameters were analyzed in accordance with the latest international recommendations for cardiac chamber quantification [21] and diastolic function evaluation [22].

### 2.4. CMR Imaging Acquisition and Analysis

CMR scans were performed with a 1.5-T magnetic resonance imaging unit (Avanto; Siemens Healthcare, Erlangen, Germany). The patients were scanned 10.1 days (interquartile range (QR), 6.5–12.8) after their cardiac arrest. The protocols included cine CMR, T1-weighted imaging before and after contrast, and T2-weighted imaging. Cine images were acquired using a steady-state free precession sequence in the horizontal long- and short axis, 2-, 3-, and 4-chamber views of the left ventricle. The imaging parameters were as follows: Repetition time (TR)/echo time (TE), 2.9/1.4 ms; flip angle, 60°; 25 phases; in-plane spatial resolution, 2.0 × 2.0 mm; slice thickness, 8 mm; slice gap, 2 mm; bandwidth 2560 Hz/pixel; acquisition matrix, 152 × 150; and field-of-view (FOV), 300 × 300 mm. Conventional breath T1-weighted images were acquired in the same long- and short axis views. The imaging parameters were as follows: TR/TE, 2.4/1.2 ms; flip angle, 20°; in-plane spatial resolution, 2.2 × 2.2 mm; slice thickness, 8 mm; bandwidth, 1080 Hz/pixel; acquisition matrix, 156 × 156; and FOV, 340 × 340 mm. Myocardial edema imaging was performed using breath-hold T2-weighted imaging in cardiac short-axis orientation. The imaging parameters were as follows: TR/TE, 2.4/1.2 ms; flip angle, 35°; in-plane spatial resolution, 2.2 × 2.2 mm; slice thickness, 8 mm; acquisition matrix, 160 × 156; and FOV, 340 × 340 mm. Late gadolinium enhancement was obtained after intravenous administration of 0.1 mmol/kg gadoxetic acid (Primovist; Bayer). The imaging parameters as follows: TR/TE, 6.2/3.0 ms; flip angle, 25°; in-plane spatial resolution 1.5 × 1.5 mm; slice thickness 8 mm; acquisition matrix, 200 × 200; and FOV, 300 × 300 mm.

All the studies were analyzed by the board-certified radiologist on duty. All the hospital has similar methods for analyzing images. The imaging findings were interpreted by 2 investigators. The CMR scans that did not show any specific findings were considered “normal”. Imaging findings that could not contribute to the diagnosis, such as myocardial wall edema related to CPR or wall dyskinesia not related to ischemic cardiomyopathy, were regarded as “non-diagnostic”.

### 2.5. Final Clinical Diagnosis

We defined final clinical diagnosis as the cause of cardiac arrest made by the medical team treating the patients including cardiologist based on all clinical information available (imaging, coronary angiography, electrophysiological study). This did not need to coincide with the final CMR diagnosis.

### 2.6. Statistical Analysis

Continuous variables are reported as median and IQR, and categorical data are presented as absolute frequencies (*n*) and percentages. We used the SPSS version 21 software for the statistical analyses.

## 3. Results

### 3.1. Study Population

**Figure 1:**
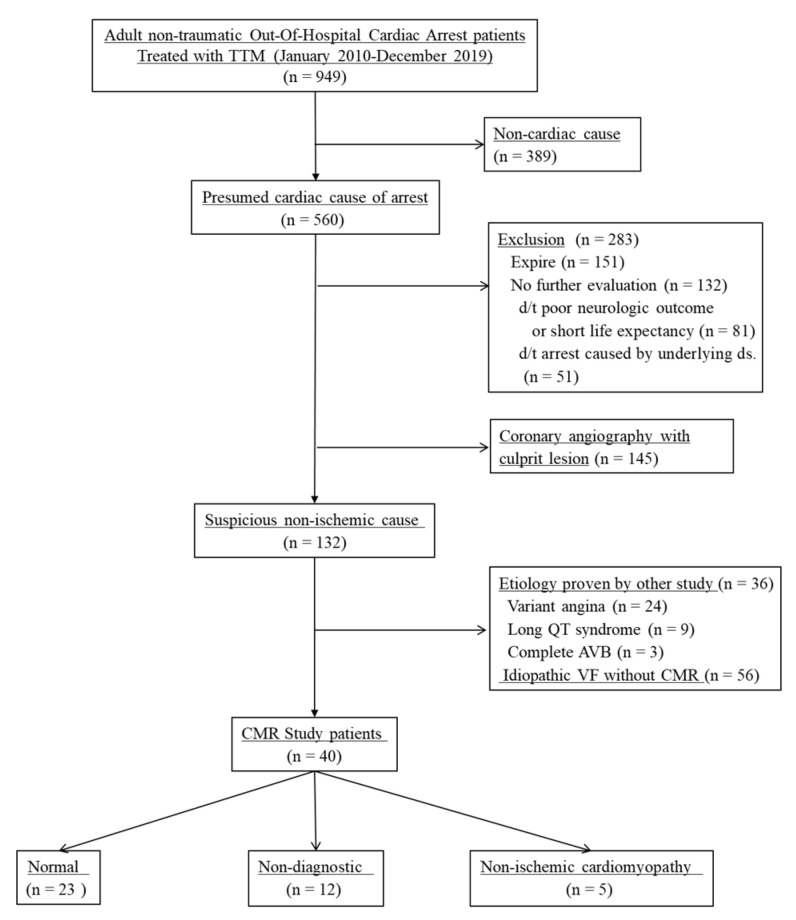
Patient enrolment flow diagram. TTM: Targeted temperature management; AVB: Atrioventricular block; VF: Ventricular fibrillation; CMR: Cardiac magnetic resonance.

Figure 1 shows the patient flow diagram of the included population. Between January 2010 and December 2019, 949 patients were included in the registry of the study facility. Of the patients, 389 with a suspected non-cardiac arrest such as drowning, hanging, and asphyxia were excluded. Among 560 patients with a presumed cardiac cause of arrest, 151 died. Eighty-one patients did not undergo further evaluation because of an anticipated poor neurological outcome or short life expectancy. Fifty-one patients with an underlying cardiac disease that was suspected as the cause of the arrest did not undergo CMR imaging. One hundred forty-five patients were found to have significant coronary lesions on coronary angiography. In 36 of the 132 patients with a suspected non-ischemic cause of arrest, the etiologies included variant angina, long QT syndrome, and complete atrioventricular block in ancillary studies. Another 56 patients were diagnosed as having idiopathic ventricular fibrillation without CMR imaging. Finally, 40 patients underwent CMR imaging. The number of patients from each hospital included in final analysis is as follows: 18, 18, and 4, respectively.

### 3.2. Baseline Characteristics

The demographic and clinical data of the patients are presented in Table 1. The median age was 37.5 years (IQR, 29.0–51.3). Of the patients, 29 (72.5%) were men and 28 (70.0%) were previously healthy. Only 1 patient experienced a cardiac arrest with an unproven cause in a previous imaging study, including coronary angiography. None of the patients had a previous myocardial infarction or heart failure. History of angina was reported in 3 patients; however, they did not perform any coronary intervention because of mild stenosis. Another 4 patients had a history of arrhythmia, which was non-life threatening. Four patients reported hypertension and 2 patients reported diabetes mellitus.

Cardiac arrest was witnessed in 33 cases (82.5%). Thirty patients (75.0%) received a bystander CPR. In 29 cases (72.5%), ventricular fibrillation was reported as the initial rhythm at the scene. Shock was performed in 33 patients (82.5%). Among the patients, 36 (90.0%) achieved spontaneous circulation at the initial presentation. Two patient had ventricular fibrillation. Pulseless electrical activity and asystole were observed in only one patient, respectively.

Median heart rate was 100 beats/min (IQR, 89–107.5). QTc duration was 469.5 mm/s (IQR, 452–489.8). ST segment change were observed in 11 patients (27.5%). LBBB and RBBB were observed in 2 (5.0%) and 4 patients (10.0%), respectively.

The time from cardiac arrest to CMR imaging varied depending on the patient’s clinical status and physician’s decision. The median time was 10.1 days (IQR, 6.5–12.8).

### 3.3. Echocardiography Analysis

The echocardiographic data, including left ventricle volume, ejection fraction, diastolic function, and presence of pericardial effusion, are shown in Table 2. The median LVEF (%) of the patients was 59.5 (54.3–63.0). The median LV EDV and ESV indexes (ml/m2) were 58.9 (45.0–65.3) and 24.2 (16.5–29.8), respectively. The median LV mass index (g/m2) was 88.2 (77.1–110.2). Diastolic dysfunction grades 1 and 2 were reported in 11 (27.5%) and 3 patients (7.5%), respectively, and another 26 patients (65.0%) showed normal diastolic functions. None of the patients showed a pericardial effusion.

### 3.4. CMR Imaging Findings and Final Diagnosis and Implantable Cardioverter Defibrillators Insertion

**Figure 2:**
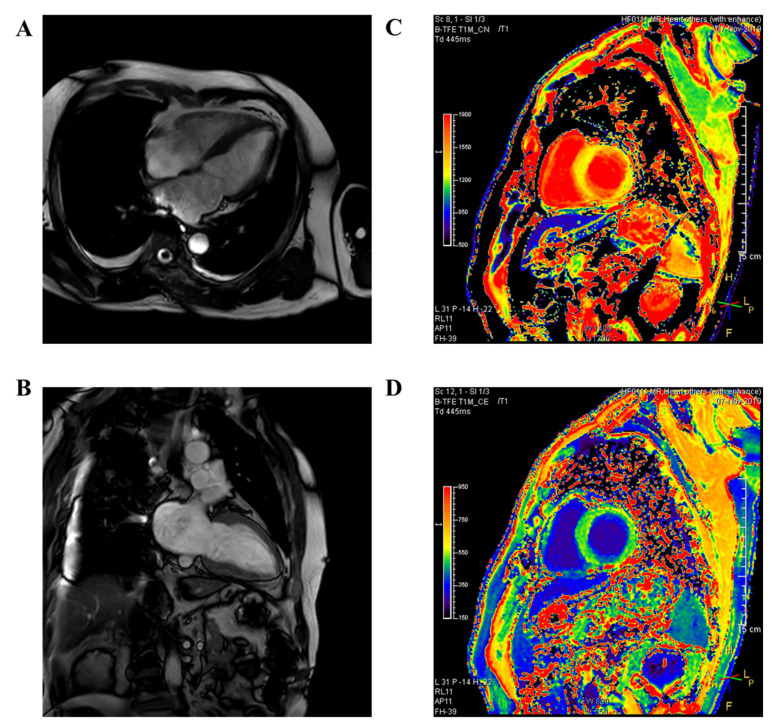
CMR scan suggesting non-ischemic cardiomyopathy. (**A**,**B**) Cine view (4 and 2 chambers). (**C**) Pre-T1 map. The T1 value is diffusely increased in the LV wall, except the lateral and inferior walls. (**D**) Post-T1 map. The T1 value is diffusely decreased in the LV endocardium.

The CMR imaging findings, final diagnoses, and implantable cardioverter defibrillator (ICD) use of the patients are presented in Table 3. Of the patients, 23 (57.5%) had normal CMR imaging findings. We observed non-diagnostic finding such as CPR related finding, wall motion dyskinesia, and decreased contractility in 12 (30%) patients. Only five patients showed the CMR imaging findings, which suggests non-ischemic cardiomyopathy. Two patients were suspected of stress-induced cardiomyopathy. Another patient showed an abnormal finding, which suggests hypertrophic cardiomyopathy. One patient showed subtle myocardial fibrosis at the right ventricle insertion point, which suggests non-ischemic cardiomyopathy. Another patient showed an increased pre-T1 value in the LV wall except the apical lateral and inferior walls; otherwise, the post-T1 value was decreased in the LV endocardium, which suggests non-ischemic cardiomyopathy (Figure 2) Table S1 presents a detailed description of the CMR imaging finding, final diagnosis, and ICD insertion status.

In patients with normal CMR findings, 14 patients were finally diagnosed as idiopathic ventricular fibrillation (VF), and six patients were diagnosed as variant angina. Other diagnoses were present in three patients. Final diagnoses of patients with non-diagnostic CMR findings were idiopathic VF in five patients, variant angina in three patients, and others in four patients. The CMR finding, which suggests non-ischemic cardiomyopathy, did not support the final diagnosis. Idiopathic VF was diagnosed in two patients, and variant angina was another diagnosis in one patient. Other diagnoses were long QT syndrome and cardiac arrest caused by bronchial edema.

## 4. Discussion

CMR imaging is an emerging tool to investigate non-ischemic cardiomyopathies and cardiac systemic disease. To our knowledge, this is the first study to investigate the usefulness and clinical impact of CMR imaging in TTM-treated patients with OHCA. CMR imaging was performed in 40 patients treated with TTM after OHCA, which revealed normal findings in 23 patients and non-diagnostic findings in 12. An abnormal finding that suggested non-ischemic cardiomyopathy, although it did not support final diagnosis, was found in five patients.

The result of this study contradicts the previous reports that support role of CMR imaging in cardiac arrest. Recent study with 164 survivors of fatal arrhythmias reported that CMR contributed to the diagnosis in nearly half of patients [11]. Moreover, among 110 OHCA survivors with inconclusive coronary angiogram, Baritussio et al., found that CMR imaging provided a clinical impact in 76 patients (70%) and contributed to the diagnosis in 27 patients (25%) [23]. Additionally, Mavrogeni et al., reported that CMR can be of great value in evaluation of arrhythmogenesis in sudden cardiac death of healthy athletes especially by its ability to differentiate non-ischemic cardiomyopathy with athlete’s heart [10]. However, another study with 36 cardiac arrest survivors found only minor findings in CMR imaging and other study that evaluated the cause in 43 cardiac arrest survivors reported that >50% of the patients who underwent CMR imaging had normal or non-diagnostic findings [4]. Considering the conflicting results of CMR imaging in the cardiac arrest survivors, the usefulness of CMR imaging is still uncertain.

An explanation for our result is the difference in study population. Previous studies included heterogeneous group of patients from non-sustained ventricular tachycardia to a history of resuscitated cardiac arrest [11,24]. However, patients treated with TTM have different characteristics to these study populations. Although TTM itself would not influence imaging quality of CMR and structural change in heart, the characteristics of patients and the duration of treatment may lessen the diagnostic ability of CMR.

In general, cardiac etiology accounts for 70% of OHCA [25,26,27], otherwise in this study about half of patients were excluded due to non-cardiac etiology. As TTM is indicated in comatose patients after OHCA, patients who recovered alert after arrest were excluded, which generally caused by cardiac etiology. If CMR had been performed in all OHCA survivors, better results would be expected. However, in other words, it could imply that proper selection of patients whom to perform CMR is needed for maximizing the ability of CMR.

In our study, idiopathic ventricular fibrillation was diagnosed in 19 patients, which accounts for approximately 10% of the total TTM patients. This is a slightly higher incidence than those reported in previous studies, which ranged from 3% to 5% [28,29]. In this study, CMR imaging was only performed in patients with good neurological outcomes. Considering the excellent survival rate in the patients without any identified heart disease [30], the possibility of structural heart disease could be low in the CMR imaging study group after TTM.

The median time from cardiac arrest to CMR imaging was 10.1 day. One study which evaluated 44 OHCA survivor who underwent coronary angiography reported that early CMR imaging performed within 1 week after cardiac arrest contributed to the diagnosis and provided prognostic implications [31]. As CMR imaging requires the patient’s cooperation, clinical status was an absolutely important requisite to perform CMR imaging. Aside from the 3 days of TTM, several days are often required to stabilize the hemodynamic status and wean the patient from ventilator support. Considering the importance of early investigation, CMR imaging would be less useful in TTM patients than in healthy sudden cardiac death survivors.

Although the recent guideline emphasizes the identification of the cause of arrest, the stepwise approach to secondary prevention after TTM is not well established [2]. In our study, three patients were diagnosed as having variant angina by ergonovine stress coronary angiography after CMR imaging. Considering the higher incidence of vasospasm in East Asians than in Caucasians, performing CMR imaging after ergonovine stress test would be reasonable for post-cardiac arrest East Asian patients [32,33].

The recent guideline emphasizes prompt initiation of TTM is necessary for CPCR survivors to ensure optimal functional and neurological outcome [34]. All the patients who do not follow commands after cardiac arrest are indicated for TTM regardless of initial rhythm. As the more patients are indicated for TTM after cardiac arrest, it would increase that performing CMR in CPCR survivor treated with TTM. This study has an advantage as preliminary study to find the diagnostic impact of CMR in the potential population. This study could provide important information to investigate the ideal time and suitable patients for CMR imaging, further study will be needed.

This study has several limitations. First, the sample size of the study was relatively small, so its results may not necessarily reflect general populations. Second, it has selection bias. CMR imaging was not performed for all the TTM patients. Many patients were excluded because of poor neurological outcome or short life expectancy. Unless neurologic recovery is guaranteed, it is not practical to perform CMR because it is expensive and not always available. Patients who were diagnosed with idiopathic VF without CMR imaging were also excluded in this study. Although cardiac MR has diagnostic advantage on sudden cardiac death to exclude structural heart disease, it is not mandatory. CMR imaging could possibly identify the etiology of cardiac arrest among the excluded patients. Third, patients whose arrests had a coronary pathogenesis were excluded; however, the possibility of dual pathology could not be ruled out. Fourth, the time and procedure of CMR imaging were not controlled in the enrolled patients. As CMR imaging is a relatively time-consuming modality and requires the patient’s cooperation for imaging quality, controlling the timing was difficult, as it depends on the patient’s clinical status. Some patients took >1 month after cardiac arrest to undergo CMR imaging, so the CMR imaging findings may not be conclusive.

## 5. Conclusions

In this study, CMR imaging may not be significantly useful for identifying unknown causes of cardiac arrest in OHCA survivors treated with targeted temperature treatment without definitive diagnosis even after coronary angiography, echocardiography, and electrophysiology studies. However, given that the study was limited by sample size and not statistically assess interaction, our results can only be viewed as hypothesis generating. Further large-scale studies will be needed to confirm these findings.

## Figures and Tables

**Table 1:** Baseline demographic and clinical data of the study population.

Characteristics	Study Population (*n* = 40)
Age (median), years	37.5 (29.0–51.3)
Male sex	29 (72.5)
Medical history	
Previously healthy	28 (70.0)
Previous cardiac arrest	1 (2.5)
Previous myocardial infarction	0 (0)
Angina	3 (7.5)
Arrhythmia	4 (10.0)
Heart failure	0 (0)
Hypertension	4 (10.0)
DM	2 (5.0)
Witnessed	33 (82.5)
Bystander CPR	30 (75.0)
Initial rhythm at scene, VF	29 (72.5)
EMS shock at scene	33 (82.5)
Initial rhythm at the emergency department	
ROSC	36 (90.0)
VF	2 (5.0)
PEA	1 (5.6)
Asystole	1 (2.5)
Electrocardiographic findings	
Rate, beats/min	100 (89–107.5)
QRS duration, mm/s	97.5 (90.3–107.5)
QTc duration, mm/s	469.5 (452–489.8)
ST segment change	11 (27.5)
LBBB	2 (5.0)
RBBB	4 (10.0)
Time from cardiac arrest to CMR imaging (median), days	10.1 (6.5–12.8)

Values are expressed as median with interquartile range or number (%). DM: Diabetes mellitus; CPR: Cardiopulmonary resuscitation; VF: Ventricular fibrillation; EMS: Emergency medical service; ROSC: Return of Spontaneous Circulation; PEA: Pulseless electrical activity; CMR: Cardiac magnetic resonance; LBBB: Left bundle branch block; RBBB: Right bundle branch block.

**Table 2:** Echocardiographic analysis.

Characteristics	Study Population (*n* = 40)
LV EDV index, mL/m^2^	58.9 (45.0–65.3)
LV ESV index, mL/m^2^	24.2 (16.5–29.8)
LV mass index, g/m^2^	88.2 (77.1–110.2)
LVEF, %	59.5 (54.3–63.0)
Diastolic function	
Normal	26 (65.0)
Diastolic dysfunction grade 1	11 (27.5)
Diastolic dysfunction grade 2	3 (7.5)
Diastolic dysfunction grade 3	0 (0)
Pericardial effusion	0 (0)

Values are expressed as median with interquartile range or number (%). LV: Left ventricle; EDV: End-diastolic volume; ESV: End-systolic volume; EF: Ejection fraction.

**Table 3:** Cardiac magnetic resonance imaging findings and final diagnosis in study population.

Finding		Final Diagnosis	
Normal	23 (57.5)		
		Idiopathic VF	14 (60.9)
		Variant angina	6 (26.1)
		Other *^2^	3 (13.0)
Non-diagnostic	12 (30)		
CPR related finding	3 (25)	Idiopathic VF	5 (41.7)
Wall motion dyskinesia	4 (33.3)	Variant angina	3 (25)
Decreased contractility	5 (41.7)	Other *^3^	4 (33.3)
Non-ischemic cardiomyopathy	5 (12.5)		
SCMP	2 (40)	Idiopathic VF	2 (40)
HCMP	1 (20)	Variant angina	1 (20)
Other finding that suggests non-ischemic cardiomyopathy *^1^	2 (40)	Other *^4^	2 (40)

Values expressed as *n* (%). VF, ventricular fibrillation; CPR, cardiopulmonary resuscitation; SCMP, stress-induced cardiomyopathy; HCMP, hypertrophic cardiomyopathy. *^1^: Diffusely increased pre T1 value in LV wall, except apical lateral and inferior wall, diffuse decreased post T1 value in LV endocardium; Subtle myocardial fibrosis at RV insertion point. *^2^: Long QT syndrome (2), WPW syndrome (1). *^3^: Long QT syndrome, coronary spasm d/t tight stenosis, Brugada syndrome, SCMP. *^4^: Long QT syndrome, Cardiac arrest d/t bronchial edema.

## Data Availability

The information contained in the database may be accessible after contacting the corresponding author and after its reasoned justification.

## Supplementary Materials

The following are available online at https://www.mdpi.com/2077-0383/10/4/794/s1, Table S1: Detailed description of cardiac magnetic resonance imaging finding, final diagnosis, and ICD insertion status in Study Population.
